# Evaluation of rockfish conservation area networks in the United States and Canada relative to the dispersal distance for black rockfish (*Sebastes melanops*)

**DOI:** 10.1111/eva.12115

**Published:** 2013-11-04

**Authors:** Katie E Lotterhos, Stefan J Dick, Dana R Haggarty

**Affiliations:** Department of Zoology, University of British ColumbiaVancouver, BC, Canada

**Keywords:** conservation genetics, effective population size, fisheries management, marine protected areas, MIGRATE, ONeSAMP, population genetics – empirical, reserve design

## Abstract

Marine reserves networks are implemented as a way to mitigate the impact of fishing on marine ecosystems. Theory suggests that a reserve network will function synergistically when connected by dispersal, but the scale of dispersal is often unknown. On the Pacific coast of the United States and Canada, both countries have recently implemented a number of rockfish conservation areas (RCAs) to protect exploited rockfish species, but no study has evaluated the connectivity within networks in each country or between the two countries. We used isolation-by-distance theory to estimate the scale of dispersal from microsatellite data in the black rockfish, *Sebastes melanops*, and compared this estimate with the distance between RCAs that would protect this species. Within each country, we found that the distance between RCAs was generally within the confidence intervals of mean dispersal per generation. The distance between these two RCA networks, however, was greater than the average dispersal per generation. The data were also consistent with a genetic break between southern Oregon and central Oregon. We discuss whether additional nearshore RCAs in southern Oregon and Washington would help promote connectivity between RCA's for shallow-water rockfishes.

## Introduction

Marine reserve or marine protected area networks are intended to protect a species or a suite of species from overexploitation. In contrast to most terrestrial species, many marine species have a bipartite life cycle in which pelagic larvae have the potential to disperse long distances by ocean currents before settling into a benthic (and sometimes sedentary) adult phase. As a consequence of this life history, results from terrestrial reserves do not provide useful principles for the design of marine reserves (Carr et al. 2003). A central issue in the design and value of marine reserve networks is the scale of dispersal relative to reserve size and configuration (Gaines et al. 2010).

Many factors are important in the efficacy of a reserve network to produce a synergistic effect on population growth, but generally the distance and direction of larval dispersal is a primary issue because it determines the rates of self-recruitment, plays a role in maintaining a persistent network of reserves, and maximizes benefits to the fishery through recruitment subsidy (Sale et al. 2005; Gaines et al. 2010). Ideally, a marine reserve network should be designed to have multiplicative effects, such that the demographic coupling of populations in separate reserves can synergistically increase numbers both within reserves (i.e., reserve connectivity) and outside (i.e., reserve subsidy to the fishery) (Gaines et al. 2010). We refer to the demographic exchange of migrants between reserves as connectivity (the source-to-destination matrix of settlers to a series of subpopulations that comprise a metapopulation connected through larval dispersal). Connectivity is determined by the larval dispersal kernel (the two-dimensional distribution of larval settlement originating from a single-source population) (definitions of connectivity and dispersal from Leis et al. 2013). Assuming no contributions from fished areas, the efficacy of a reserve network is predicted to increase asymptotically as the size of individual reserves increases relative to the mean dispersal distance, regardless of the shape of the tail of the dispersal distribution (Botsford et al. 2001; Lockwood et al. 2002).

Although the amount of connectivity among reserves is an important factor in the design of a network, many reserve networks are designed without knowledge of the scale of dispersal for the protected species. Estimating connectivity is problematic in marine species with long pelagic larval stages that have potential for long-distance dispersal (Roughgarden et al. 1988; Mora and Sale 2002). Historically, it was assumed that self-recruitment was insignificant because of long-distance dispersal via these pelagic larvae. Recent studies of tropical reef fish have found, however, direct evidence for self-recruitment through the use of chemical tags (Jones et al. 1999, 2005), isotopic tracers (Almany et al. 2007), otolith trace-element analysis (Swearer et al. 1999; Warner et al. 2005; Standish et al. 2008), and genetic parentage analysis (Jones et al. 2005; Planes et al. 2009; Saenz-Agudelo et al. 2009; Christie et al. 2010), suggesting that self-retention is greater than previously thought. Inference from these studies is limited, however, because they only estimate the amount of self-recruitment to a single location – not the amount of connectivity between populations (but see Planes et al. 2009; Christie et al. 2010). Although connectivity between marine populations is inherently stochastic on short timescales (Siegel et al. 2008), average connectivity may be more relevant to demographics because it describes the buildup of multiple age classes over time (Gaines et al. 2010).

In this study, we evaluate connectivity between recently established rockfish conservation areas (RCAs) in Canada (Yamanaka and Logan 2010) and in the United States. The rockfishes (genus *Sebastes*) are a speciose genus consisting of over 100 species, most of which occur in the northeast Pacific (Love et al. 2002). Many rockfish species are long-lived (30–100+ years) and suffered severe declines in the 1980s as a result of heavy fishing pressure (Hilborn et al. 2004). Because the longevity of rockfish makes them slow to recover from overfishing, they have been the subject of conservation efforts for the last decade. As these RCA's have all been established relatively recently, there is still a paucity of information on their role in the persistence and recovery of rockfish populations. Rockfishes are managed in the United States and Canada as a high-value fishery. In Canada, rockfish are managed by the Department of Fisheries and Oceans. In the United States, they are managed by state agencies in inshore waters, by the Pacific Fisheries Management Council in the offshore waters of California, Oregon and Washington, and by the National Oceanic and Atmospheric Administration (NOAA) in the offshore waters of Alaska (http://www.pcouncil.org/).

In addition to mean dispersal, reserve design should take into account retention zones, oceanographic conditions, and major currents to maximize biological exchange among reserves (Murray et al. 1999; Botsford et al. 2003). In rockfishes, connectivity and gene flow has been shown to be influenced by possible barriers to dispersal such as retention zones and mesoscale eddies that entrain planktonic larvae (Parrish et al. 1981; Morgan and Botsford 1998; Wing et al. 1998; Hyde and Vetter 2009; Hess et al. 2011). Connectivity may also be influenced by life-history traits (timing and depth of egg or larval release, larval swimming ability, homing behavior, and pelagic duration) that interact to determine the extent to which oceanographic events and currents affect the scale of dispersal (Sponaugle et al. 2002; Galarza et al. 2009). On the Pacific Coast of the United States and Canada, major oceanographic features include upwelling jets that occur in the spring and summer. Upwelling fronts occur when northerly winds drive water masses offshore that are replaced by cold, nutrient-rich water that is upwelled from depth. The Pacific Coast can be divided into four major upwelling centers (Parrish et al. 1981). These upwelling cells may play an important factor in dispersal and recruitment for several fish and invertebrate species (Morgan and Botsford 1998; Wing et al. 1998; Bjorkstedt et al. 2002), and transitions in upwelling regimes may coincide with barriers to dispersal in rockfishes (Hyde and Vetter 2009; Hess et al. 2011).

Information on the relationship among dispersal distance, oceanographic features, and reserve design is of central importance to the evaluation of RCAs in the United States and Canada. We evaluated the population genetic structure of the black rockfish, *Sebastes melanops* from southern Oregon to northern British Columbia (BC) and used isolation-by-distance theory to estimate the scale of dispersal. Isolation by distance (IBD) is a phenomenon in which genetic distance between populations increases with geographic distance between them. The IBD slope can be used in combination with information about the effective density to estimate the scale of dispersal (Rousset 1997). Using IBD to estimate dispersal is attractive because it is less affected by historically rare dispersal events and only requires the distance between the samples rather than knowledge of population genetic boundaries (Rousset 1997; Hardy and Vekemans 1999). Recently, this approach has been used to estimate mean dispersal in organisms as diverse as skinks (Sumner et al. 2001), damselflies (Watts et al. 2007), and marine fishes (Puebla et al. 2009, 2012; Pinsky et al. 2010; Palof et al. 2011; Lotterhos 2012).

We compared the genetic estimate of dispersal to the spacing between RCAs that would protect black rockfish in the United States and Canada and found that a large spatial gap existed between nearshore RCAs in the United States and Canada, potentially reducing connectivity between reserve networks in these countries. We also compared the estimate of effective density from genetic data to the census density from SCUBA and found that the effective density was substantially lower compared to census density. Our results have implications for the black rockfish populations that comprise an important nearshore recreational fishery in both countries (Wallace et al. 2008), and other rockfish species with similar life histories.

## Materials and methods

### Study system and species

**Figure 1:**
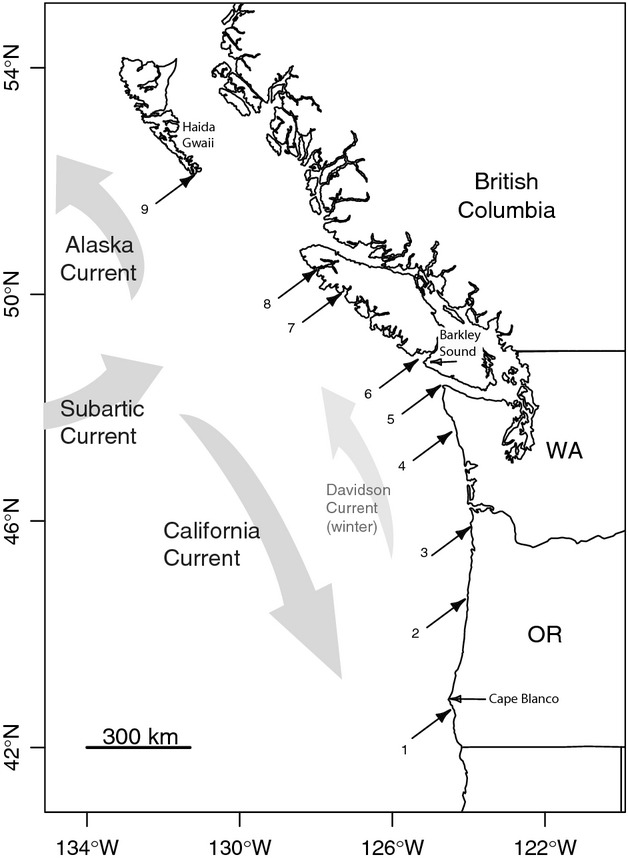
Locations of the nine sites sampled for adult *Sebastes melanops* along the west coast of the United States and Canada. Information on sample sizes and collection year for each sample location can be found in Table 1. Arrows indicate directions of the major currents. The Subarctic Current bifurcates between 45–50°N and 130–150°W, resulting in variable currents in that region.

Our research area spans the outer coast from southern Oregon in the United States to northern BC (Fig. 1). Black rockfish are typically found in high-exposure areas and tend to form schools associated with kelp or over high-relief habitats (Love et al. 2002). Our sampling does not include the inner waters of the Salish Sea (Strait of Georgia, Puget Sound, the Strait of Juan de Fuca) nor Johnstone Strait. Abundance of *S. melanops* is very low in these areas (Williams et al. 2010). Indeed, genetic differentiation has been found between these inner waters and the outer coast in some rockfish species (Berntson and Moran 2009), indicating that patterns of dispersal may differ between the inner waters and outer coast.

In published studies, the depth distribution of *S. melanops* was between 1 and 48 m, with a mean of 16 m (Johnson et al. 2003; ROV surveys) or 31 m (Parker et al. 2007; tagging study), although they may be found as deep as 55 m (Love et al. 2002). Typically, adults have small home ranges of 418 m in radius, although home ranges may be ephemeral and fish may relocate over a few kilometers (Parker et al. 2007).

Rockfish have a planktonic phase, and it is likely that the majority of their dispersal occurs during the larvae phase. Rockfish have internal fertilization and mating occurs in the late fall (October–November). Female rockfish store sperm and use that sperm to fertilize their eggs around December to January, and then brood those eggs for about a month before releasing from 125 000 to 1 000 000 well-eyed larvae in parturition from January to March (Love et al. 2002; Berkeley et al. 2004a). Rockfish larvae are capable swimmers shortly after parturition and can swim at speeds of 2–6 mm/s during routine activity in the laboratory (Fisher et al. 2007). The pelagic period of black rockfish larvae is 60–80 days long, after which they settle into nearshore kelp forest habitats in April and early May (Lotterhos and Markel 2012). Black rockfish larvae have high dispersal potential (100 s km) as a result of this long larval period.

The rockfish larval period spans a seasonal transition in the predominant currents. Off of the northern part of Vancouver Island at about 45–50°N and 130–150°W, the Subarctic Current splits into the northward-flowing Alaska current and the southward-flowing California current (Fig. 1, Thomson 1981; Shanks and Eckert 2005). During the winter when rockfish larvae are released, the predominate wind source is from the south and drives the northerly flowing Davidson Current, moving the California Current offshore (Thomson 1981). Usually, beginning in early April, the predominant wind source switches to the northwest and the southern-flowing California Current moves closer to shore, setting up conditions for coastal upwelling. Pelagic juveniles of black rockfish have been found inside upwelling fronts (Larson et al. 1994; Sakuma and Ralston 1995; Bjorkstedt et al. 2002), and transport of larvae from upwelling fronts into the nearshore environment is thought to occur by upwelling-relaxation events or by eddies that bud off the fronts (Sverdrup 1938; Roughgarden et al. 1988; Sakuma and Ralston 1995; Wing et al. 2003; Barth et al. 2007).

### Tissue collection, DNA extraction, and genotyping

Tissues from adult *S. melanops* were collected between 2005 and 2010 from several locations between southern Oregon and northern BC (Fig. 1). Samples that were collected within 50 km of each other were pooled for analysis, leading to a total of nine sampled populations. We found that this pooling strategy reduced variance around the IBD relationship but did not affect the slope. Fin clips were taken from either commercial or recreational fishermen, or from fish caught by hook-and-line and returned to the wild. Fin clips were stored in 95% ethanol or silica gel beads (Garden Medicinals and Culinaries #8113). In all, 572 adults were genotyped, and sampling information for each location, including sample size and year of collection, is shown in Table 1.

**Table 1:** Information on sampling location, sample size, and time of collection for adult black rockfish. Map ID corresponds to the location marked on Fig. 1.

Map ID	State, region	Site name	Latitude	Longitude	Sample size	Year(s) of collection
1	OR, USA	Rogue Reef, Island Rock, Orford Reef	42°39.784′N	124°28.728′W	77	2005
2		Till Rock, Newport	44°37.122′N	124°05.537′W	50	2009
3		Cannon Beach	45°53.434′N	123°57.694′W	47	2009
4	WA, USA	Northwest Westport	47°34.226′N	124°26.906′W	54	2009
5		Tatoosh	48°23.479′N	124°44.340′W	35	2008, 2010
6	Vancouver Island, BC	Barkley Sound	48°50.762′N	125°19.888′W	175	2007–2010
7		Kyuquot and Checleset Bay	50°00.679′N	127°20.730′W	55	2010
8		Quatsino Sound	50°26.110′N	127°58.828′W	49	2009
9	Haida Gwaii, BC	Gwaii Haanas	52°07.217′N	131°10.353′W	30	2010

OR, Oregon; WA, Washington; BC, British Columbia.

Haida Gwaii was formerly known as the Queen Charlotte Islands.

All individuals were genotyped at eight microsatellite loci that were developed for this and other *Sebastes* species: Spi4 (GenBank accession number AY192599), Spi6 (AY192600), Spi10 (AY192603), and Spi12 (AY192604) (Gomez-Uchida et al. 2003); Sma7 (AY654599) (Wimberger et al. 1999); Sth37 (AB033427) (Sekino et al. 2000), Sme4 (AF142486), and Sme9 (AF142491). Details about the DNA extraction, PCR protocol, and multiplexing can be found in Lotterhos and Markel (2012).

### Genetic analysis: descriptive statistics

Fish caught from the same location in different years were pooled for genetic analysis because the timescale of sampling was short in comparison with the 50-year life span of black rockfish.

We calculated observed and expected heterozygosity (Nei 1978), Weir and Cockerham (1984) *F*-statistics, genotypic disequilibrium, and conformations to Hardy–Weinberg equilibrium for each locus within and across samples in FSTAT (Goudet 1995). To test the null hypothesis of genetic homogeneity among sampled populations, we estimated global and pairwise *F*_ST_ values and tested their significance with 10 000 permutations in the program FSTAT (Goudet 1995). Significance of *F*_ST_ for each locus was based on a test for population differentiation with the log-likelihood *G*-statistic with the assumption of random mating within samples, implemented in FSTAT. We controlled for the false discovery rate by comparing *P*-values for each family of tests (i.e., tests that come from the same distribution, such as tests for genotypic disequilibrium, or pairwise tests for *F*_ST_) with the Benjamini–Hochberg sequential procedure (Benjamini and Hochberg 1995).

To ensure that any significant deviations from Hardy–Weinberg equilibrium were not an artifact of null alleles, we estimated the frequency of null alleles. Traditional estimates of null allele frequencies (Dempster et al. 1977; Chakraborty et al. 1992; Brookfield 1996; Chapuis and Estoup 2007) that assume random mating and Hardy–Weinberg equilibrium will be biased upward when the true *F*_IS_ > 0 (Van Oosterhout et al. 2006; Chybicki and Burczyk 2009). While there is no reason to expect inbreeding in rockfish, it is possible that *F*_IS_ > 0 because of a Wahlund effect due to high migration and IBD structure in this species. We used the program INEST to estimate the frequencies of null alleles under an individual inbreeding model using the Gibbs sampler and 10 000 iterations (Chybicki and Burczyk 2009).

### Genetic analysis: population structure

We used the program STRUCTURE (Pritchard et al. 2000) to determine whether there were any strong patterns of genetic structure in our dataset. To ensure that our STRUCTURE results were reproducible, we followed the guidelines of Gilbert et al. (2012). The STRUCTURE analysis is detailed in the Data S1.

We also performed a principal components (PC) analysis on populations in GENODIVE 2.0 (Meirmans and Van Tienderen 2004) to assess whether there was a geographic pattern of clustering among sampled populations.

### Genetic analysis: isolation by distance

We used isolation-by-distance (IBD) theory to estimate the scale of dispersal in black rockfish. The IBD slope can be used to estimate the standard deviation of the dispersal distribution or dispersal kernel, which is a function that describes the probability of dispersal at different distances from the source. For one-dimensional habitats such as coastlines, the standard deviation of the dispersal kernel (*σ*, also known as the axial parent-offspring distance) is estimated from the relationship


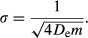
(1)

Where *D*_e_ is the effective density and *m* is the slope of the relationship between *F*_ST_/(1 − *F*_ST_) and geographic distance (Wright 1943; Rousset 1997). The effective density is the effective population size per unit distance. The quantity *σ* is affected by gene flow over several recent generations – and so it is affected by both contemporary and past connectivity (Botsford et al. 2009).

Equation 1 assumes that the population is at equilibrium between gene flow, mutation, and drift. There are some known issues with the interpretation of *σ*. Uncertainty or bias in estimating the effective density can in turn bias the estimate of *σ*. In addition, eqn 1 assumes a linear lattice of migration with an equal effective density across space, a dispersal distribution that is equal for each deme in the lattice, discrete generations, and equilibrium between gene flow and drift (Rousset 1997; Robledo-Arnuncio and Rousset 2010). Generally, though, eqn 1 has been shown to be very robust. In simulated populations with spatially fluctuating density and density-dependent regulation, the relationship holds irrespective of dispersal kurtosis (Robledo-Arnuncio and Rousset 2010). Likewise, eqn 1 is robust to leptokurtic distributions of dispersal distance (Rousset 2000) and is insensitive to different shapes of symmetrical dispersal distributions (Lee and Hastings 2006). IBD theory is robust to dispersal distributions of different shapes, including those that include skew or kurtosis, because these higher moments are ignored in the diffusion approximation (Rousset 1997). Moreover, the IBD slope is robust to the high mutation rates of microsatellite loci, and high heterozygosity of microsatellite markers (0.7–0.8) can substantially increase the precision of the estimation (Leblois et al. 2003). A recent simulation study in reef fish showed that a nonsignificant IBD relationship with lots of scatter can still accurately reflect the scale of dispersal (Puebla et al. 2012).

### Estimation of IBD slope

A Mantel test was used to test the null hypothesis of no relationship between pairwise geographic distance and pairwise genetic differentiation (Mantel 1967). Pairwise geographic distance was measured as shortest along-shore route between sampling sites. Pairwise genetic distance between sampling sites was measured as *F*_ST_/(1 − *F*_ST_). We implemented the Mantel test with 999 iterations in R version 3.0.1 (R Core Development Team 2009). We used reduced major axis (RMA) regression to estimate the slope and confidence intervals of genetic distance as a function of geographic distance. We chose RMA because Ordinary Least Squares Regression (OLS) assumes the independent variable (in this case, number of genetic steps separating populations) is measured without error. While this holds true for simulated data, it does not hold for empirical data, where this distance between sites is an approximation to the number of genetic steps between them (Hellberg 1994). When error exists in an independent variable, OLS will underestimate the true regression slope (McArdle 1988). In addition, confidence intervals in the RMA are computed by permutation (similar to a Mantel test) and should therefore be more robust to nonindependence among samples.

As IBD theory assumes an equilibrium between gene flow, mutation, and drift (Rousset 1997; Robledo-Arnuncio and Rousset 2010), we wanted to determine the extent to which our data conformed to this assumption. Approximately 14 000 years ago, the Cordilleran ice sheet would have covered most of the coastal populations that we sampled north of Washington (Clague and James 2002). It is possible that despite high dispersal and a generation time of 7–10 years, an equilibrium has not yet been reached. We tested for equilibrium in two ways. First, we tested for the constancy of the IBD slope over geographic distance as recommended by Bradbury and Bentzen (2007). We divided the samples into two groups that spanned approximately 600–700 km (half of the geographic span of all samples) and examined the IBD slope in each group. Second, we also examined correlations between latitude and allelic richness, or latitude and *H*_e_, because a decrease in allelic richness or *H*_e_ with latitude may indicate that the population has not yet reached equilibrium since expansion from the last glacial maximum.

### Estimation of effective density

Effective density is the effective population size per unit area. We attempted to estimate the effective population size with three methods: by linkage disequilbirum (LDNe, Waples and Do 2008), by approximate Bayesian computation (ONeSAMP, Tallmon et al. 2008), and by the coalescent (MIGRATE, Beerli and Felsenstein 2001; Beerli 2006). The first two methods estimate a contemporary population size, while the third method estimates an ancestral population size. For each method, we estimated *N*_e_ for each of the nine sampled populations, and for a combined sample of all 572 individuals. Under the high migration rates that could occur in black rockfish populations, the value of *N*_e_ estimated from a subpopulation will be larger than the true *N*_e_ of that subpopulation because of gene flow and will probably be closer to the metapopulation *N*_e_ (Waples and England 2011). Therefore, we used the mean of the finite *N*_e_ estimates as an estimate of metapopulation *N*_e_ for each estimator.

LDNe uses the Burrows method to estimate *N*_e_ from linkage disequilibrium in the dataset (Waples 2006; Waples and Do 2008). We estimated *N*_e_ in LDNe excluding alleles with a frequency <5%.

ONeSAMP calculates eight summary statistics from a microsatellite dataset and uses approximate Bayesian computation to infer *N*_e_ from 50 000 simulated datasets (Tallmon et al. 2008). We implemented ONeSAMP with a prior on *N*_e_ from 2 to 10 000 (the minimum and maximum values possible for the program).

The coalescent method in MIGRATE (Version 3.2.7) estimates *θ *= 4 *N*_e_*μ*. The MIGRATE analysis strategy estimated *θ* with Bayesian inference with mutation rate estimated independently for each locus. Proposal distributions for *θ* used slice sampling, with uniform priors from 1 to 600. We used Markov chain settings of one long chain with 5000 recorded steps (long-sample), 10 concurrent chains (replicates), and 15 000 discarded trees per chain (burn-in). Multiple Markov chains were performed with four chains at temperatures of 10^5^, 3, 1.5, and 1 with a swapping every 10 chains and an update frequency of 0.75. All other parameters were left at default values. Runs were performed at the high-performance computational facility at Florida State University. We extracted *N*_e_ from *θ* using mean mutation rates published in other fish species. We surveyed the mutation rates observed in fish in nature and found that they ranged from 9.4*10^−4^ to 9.1*10^−3^ with an average of 7.2*10^−4^ over all 61 loci surveyed (i.e., the average included loci in which the estimated mutation rate equaled 0: Jones et al. 1999; Steinberg et al. 2002; Yue et al. 2007). As it is possible that many of these studies may not have had the power to detect lower mutation rates, we assumed a lower *μ* of 1*10^−5^ and upper μ of 9.1*10^−3^. The confidence intervals for *N*_e_ calculated from *θ* included these confidence intervals on *μ* as well as the confidence intervals on posterior distribution of *θ* from MIGRATE. This interval on mutation rates in microsatellites is in line with those observed in many model species (Ellegren 2000).

We computed the mean and confidence intervals on *D*_e_ by dividing the mean and confidence intervals of *N*_e_ from each program by the span of distance covered by our sampling.

### Estimation of census density

We obtained census density estimates from Barkley Sound, BC (Fig. 1, sample 6) and used the REEF database (the Reef Environmental Education Foundation, http://www.reef.org, REEF 2013) to justify whether our density estimates from this location were representative of the entire sampling area.

In Barkley Sound, British Columbia, we visually estimated rockfish density by counting black rockfish on 30 × 3 m transects while SCUBA diving. We completed 69 transects at 31 sites in 2010 and 124 transects at 31 sites in 2011. Transects were distributed between 6 and 18 m of depth in rocky habitat.

We compared the densities observed in Barkley Sound to densities in the rest of our sampling area, as reported through the REEF volunteer surveys (REEF 2013). REEF surveys use the roving diver technique, in which divers swim throughout a dive and record every fish species that can be positively identified (Schmitt and Sullivan 1996). Each recorded species is assigned one of four abundance categories based on about how many were seen throughout the dive: single (one fish), few (2–10), many (11–100), and abundant (>100). REEF calculates a weighted density average from the number of surveys in which the species was observed, weighted by abundance category (single = 1, few = 2, many = 3, and abundant = 4). REEF also calculates a sighting frequency (%SF) as a measure of how often the species was observed across all surveys. As black rockfish are relatively common and easy to identify, we used the combined novice and expert surveys in the REEF database.

As we found similar densities in the REEF surveys across our study area, we used the mean density and confidence intervals of the Barkley Sound transects to estimate the population size of black rockfish across our study area, assuming there is 113.36 km^2^ of black rockfish habitat per 431 km of coastline (estimate from Oregon, Sampson 2007). This method of extrapolation accounts for microgeography in rockfish habitat along the coastline. We also compared this estimate of population size with the population size estimated from a mark-recapture study in Newport, OR and extrapolated across our study range (Sampson 2007).

### Estimation of *N*_e_/*N*

We estimated the *N*_e_/*N* ratio for each *N*_e_ estimator, with confidence limits calculated from the confidence intervals on both *N*_e_ and *N*.

### Estimation of reserve spacing and area in United States and Canada

We included RCAs and Marine Protected Areas (MPAs) in British Columbia, Washington and Oregon that were likely to protect black rockfish in the analysis. We read the regulations for each conservation area and included it if: (i) it was on the outer coast (the following areas in the Salish Sea were excluded: Strait of Georgia, Puget Sound, Strait of Juan de Fuca, and Johnstone Strait), (ii) it contained habitat <50 m in depth, and (iii) rockfish were protected from both commercial and recreational fishing.

In British Columbia, RCAs prohibit commercial and recreational fisheries that target or lead to by-catch of rockfish and most are within the depth range of black rockfish (Yamanaka and Logan 2010).

In Washington, the Olympic Coast National Marine Sanctuary (NMS) is a large MPA on the open coast. Black rockfish, however, are not protected from recreational or commercial long line fishing in the Olympic Coast NMS (personal communication, Liam Antrim, Olympic Coast NMS, Nov. 15, 2012) so it was also excluded from the analysis. Although there are some deep water RCA's on the Washington outer coast, no areas protect nearshore species like black rockfish.

Oregon is in the process of designating a system of nearshore MPAs and marine reserves. Black rockfish are protected from fishing in the marine reserves that prohibit all extractive activities. We included the marine reserves that are currently in place (Otter Rock and Redfish Rocks) as well as reserves that will come into effect by 2016 (Cape Falcon, Cascade Head, and Cape Perpetua) (personal communication, Keith Matteson, Oregon Department of Fish and Wildlife, February 12, 2013). Commercial fishing and/or recreational fishing are also prohibited in a number of areas designated by the National Marine Fisheries Service; however, most of these areas are offshore and do not intersect with the typical depth range of black rockfish. Areas shallower than 50 m are found in two of these Essential Fish Habitat Conservation Areas: Hecata Bank and Newport Rockpile-Stonewall Bank. Although no black rockfish have been found in drop camera studies of Hecata Bank and the Newport Rock/Stonewall Bank (personal communication, Matthew Blume, Oregon Department of Fish and Wildlife, February 15, 2013), we included them in our analysis as they contain some potential black rockfish habitat.

We plotted the RCAs and marine reserves in ArcMap 10.1 (ESRI 1999–2012) along with the 50-m contour lines using the GCS North American 1983 coordinate system and the Canada Albers Equal Area Conical projection. To map the distance between adjacent conservation areas, we created a line shapefile and drew lines or multipart lines using the ‘snap to nearest feature’ tool to find the closest edge of each conservation area. The length of each line was then calculated in kilometers using the calculate geometry tool. The number of adjacent reserves included in our calculation depended on geographic location of the focal reserve, but varied from one to three. The reserve areas and mean distance among RCAs in BC and Marine Reserves in Oregon were then compared with the estimated dispersal distance of black rockfish.

## Results

### Descriptive statistics

After the reamplification of failed PCRs, the dataset had 0.45% missing data, corresponding to 0.17–0.86% missing per locus.

Observed heterozygosity within samples for the loci ranged from 0.122 to 1 (Table 2). All sampled populations conformed to Hardy–Weinberg expectations after correcting for multiple tests. The expected frequencies of null alleles ranged from 0.8% to 2.6% and were not correlated with *F*_IS_ among loci (Table 3). No sampled population showed significant linkage disequilibrium after correcting for multiple tests.

**Table 2:** Observed and expected heterozygosity, *F*_IS_, and allelic richness (*A*_r_) per locus and per population (population numbers correspond to the map in Figure 1). Uncorrected *P*-values are shown for *F*_IS_, but no sampled population deviated significantly from Hardy–Weinberg equilibrium after correction for multiple tests.

		Population								
Locus		1	2	3	4	5	6	7	8	9
Sma7	*H*_o_	0.286	0.420	0.575	0.519	0.400	0.480	0.527	0.417	0.533
	*H*_e_	0.358	0.435	0.540	0.613	0.382	0.457	0.578	0.499	0.533
	*F*_IS_	0.203 (*P* = 0.013)	0.034	−0.064	0.154 (*P* = 0.068)	−0.046	−0.051	0.087 (*P* = 0.018)	0.165 (*P* = 0.081)	−0.001
	*A*_r_	4.398	3.000	4.633	4.879	3.000	4.752	4.869	4.619	6.000
Sme4	*H*_o_	0.935	0.980	0.894	0.926	1.000	0.943	0.873	0.878	1.000
	*H*_e_	0.937	0.949	0.957	0.955	0.947	0.951	0.950	0.949	0.959
	*F*_IS_	0.002	−0.033	0.066 (*P* = 0.046)	0.031	−0.056	0.009	0.082	0.076 (*P* = 0.034)	−0.043
	*A*_r_	19.612	20.668	24.600	23.313	18.606	22.504	22.666	22.680	22.000
Sme9	*H*_o_	0.882	0.840	0.894	0.906	0.824	0.873	0.873	0.959	0.967
	*H*_e_	0.897	0.905	0.886	0.899	0.911	0.889	0.897	0.912	0.909
	*F*_IS_	0.017	0.072	−0.008	−0.008	0.096 (*P* = 0.063)	0.018	0.027	−0.051	−0.064
	*A*_r_	16.122	16.667	13.884	15.131	18.347	15.477	16.378	15.878	16.000
Spi10	*H*_o_	0.584	0.640	0.468	0.482	0.353	0.420	0.482	0.367	0.467
	*H*_e_	0.635	0.615	0.464	0.504	0.364	0.484	0.503	0.408	0.537
	*F*_IS_	0.079	−0.040	−0.008	0.045	0.029	0.133 (*P* = 0.0028)	0.043	0.100	0.131
	*A*_r_	7.809	6.419	6.460	8.173	4.987	7.167	7.183	7.482	7.000
Spi12	*H*_o_	0.182	0.180	0.149	0.204	0.177	0.190	0.241	0.122	0.133
	*H*_e_	0.170	0.169	0.141	0.191	0.168	0.192	0.248	0.118	0.129
	*F*_IS_	−0.069	−0.063	−0.059	−0.065	−0.050	0.014	0.028	−0.038	−0.036
	*A*_r_	2.909	2.968	2.637	4.465	3.869	3.620	2.915	3.204	3.000
Spi4	*H*_o_	0.299	0.460	0.404	0.537	0.429	0.457	0.473	0.449	0.500
	*H*_e_	0.359	0.487	0.423	0.581	0.423	0.461	0.487	0.456	0.529
	*F*_IS_	0.167 (*P* = 0.015)	0.056	0.044	0.075	−0.014	0.008	0.029	0.016	0.055
	*A*_r_	7.497	8.801	8.291	10.183	7.782	9.031	7.934	9.587	11.000
Spi6	*H*_o_	0.882	0.880	0.870	0.759	0.857	0.794	0.789	0.796	0.767
	*H*_e_	0.875	0.849	0.825	0.863	0.868	0.858	0.838	0.845	0.819
	*F*_IS_	−0.007	−0.037	−0.054	0.12 (*P* = 0.029)	0.012	0.074 (*P* = 0.011)	0.059	0.058	0.064
	*A*_r_	11.284	11.388	8.746	10.031	12.265	11.450	10.512	11.756	9.000
Sth37	*H*_o_	0.584	0.500	0.532	0.574	0.546	0.531	0.455	0.653	0.333
	*H*_e_	0.524	0.513	0.515	0.502	0.535	0.521	0.505	0.519	0.475
	*F*_IS_	−0.115	0.025	−0.032	−0.143	−0.020	−0.020	0.099	−0.259 (*P* = 0.040)	0.298 (*P* = 0.10)
	*A*_r_	3.165	2.600	2.638	2.000	2.993	2.955	2.545	3.224	2.000

**Table 3:** Properties of the loci.

Locus	*A*_n_	*A*_r_	NAfreq	*F*_ST_
Sma7	9	4.5	0.013	0.010**
Sme4	32	21.8	0.008	0.002†
Sme9	28	16.0	0.006	0.001
Spi10	11	7.0	0.023	0.007***
Spi12	7	3.3	0.010	−0.002
Spi4	19	8.9	0.022	0.002*
Spi6	21	10.7	0.024	0.002***
Sth37	8	2.7	0.026	0.0005†
Overall				0.0023***

*A*_n_, allele number; *A*_r_, allelic richness; NAfreq, frequency of null alleles; *F*_ST_, the fixation index of Weir and Cockerham (1984).

Starts indicate significance levels by permutations

† (*P* < 0.1,

* *P* < 0.05,

** *P *< 0.01,

*** *P *< 0.001).

Overall, *F*_ST_ = 0.0023 was small but significant (Table 3). Several pairwise tests for *F*_ST_ were significant after the Benjamini–Hochberg correction, and these were between Orford Reef in Oregon (pop. 1) and all other samples, as well as between Newport, Oregon (pop. 2) and the most northern samples (Table 4).

**Table 4:** Pairwise *F*_ST_ (Weir and Cockerham 1984, below diagonals) and corresponding *P*-values (above diagonals) between sampled populations.

Population	1	2	3	4	5	6	7	8	9
1		0.00278*	0.00278*	0.00139**	0.00139**	0.00139**	0.00139**	0.00139**	0.00139**
2	0.0025*		0.24861	0.30833	0.03889	0.08889	0.02361	0.00694*	0.00278*
3	0.0063*	0		0.8375	0.1625	0.7625	0.37222	0.94028	0.14028
4	0.0116**	0.0015	−0.0008		0.475	0.59722	0.81111	0.98333	0.06944
5	0.0075**	0.0033	−0.0008	0.0038		0.58194	0.12917	0.70833	0.26944
6	0.0051**	−0.0001	−0.001	0.0027	−0.0017		0.12639	0.52639	0.01667
7	0.0091**	0.0007	0.0004	−0.0021	0.003	0.0019		0.33472	0.14861
8	0.0106**	0.0043*	−0.0022	−0.0005	−0.0031	0.0007	0.0008		0.56667
9	0.0137**	0.0017*	0.0037	0.0026	0.0027	0.0039	−0.0003	−0.0004	

Asterisks indicate significance levels after the Benjamini–Hochberg correction for multiple tests (^†^*P* < 0.1,

* *P* < 0.05,

** *P *< 0.01, ^***^P < 0.001).

### STRUCTURE and PCA results

The STRUCTURE analysis indicated a general lack of strong genetic structure in the dataset (results are detailed in Data S1).

**Figure 2:**
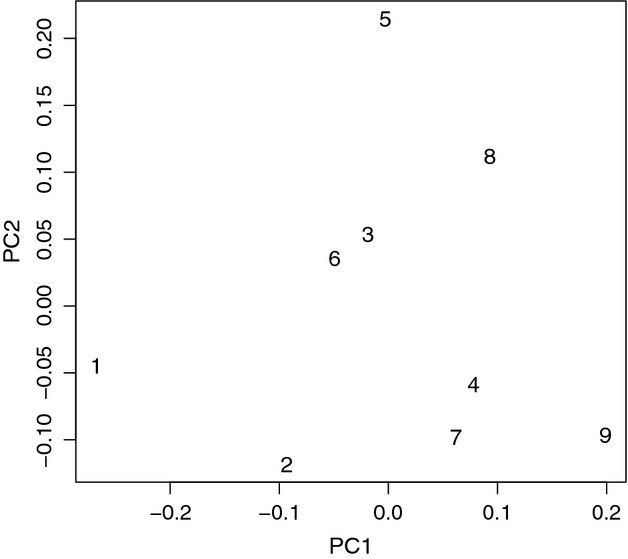
Results of the principal components (PC) analysis for populations conducted in GENODIVE 2.0. The first PC axis explained 28.5% of the variance (eigenvalue 0.018) and the second PC axis explained 20% of the variance (eigenvalue 0.013).

The first two axes of the PC analysis explained approximately 49% of the total variance. The first PC axis explained 28.5% of the variance (eigenvalue 0.018) and separated populations approximately according to latitude (Fig. 2). The second PC axis explained another 20% of the variance (eigenvalue 0.013) and separated populations in the middle of the range. Population 1 (samples collected near Port Orford, OR) was a clear outlier in the PCA (Fig. 2).

### Isolation by distance and tests for equilibrium

**Figure 3:**
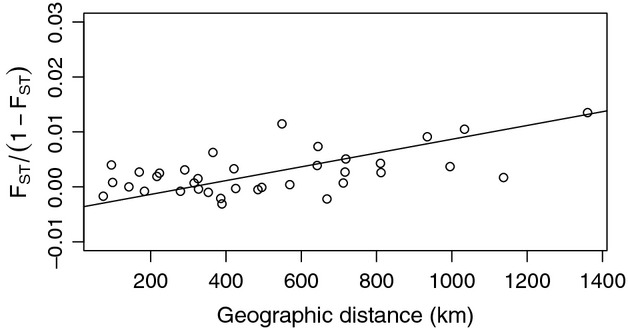
Genetic distance as a function of geographic distance for all sampled sites.

Across all sampled populations, we observed a significant correlation between geographic distance and genetic distance (Mantel test *r *=* *0.57, *P* = 0.005) and a significant IBD slope RMA slope = 1.25*10^−5^ with 95% CI in (7.35*10^−6^, 2.15*10^−5^), (Fig. 3). To determine whether the population was at or near equilibrium, we (i) examined the IBD slope on smaller spatial scales and (ii) analyzed correlations between latitude and allelic richness and latitude and *H*_e_. We found positive IBD slopes at each end of the range, with a slightly higher slope in the south [samples 1–5: Mantel correlation = 0.73, Mantel *P* = 0.02, RMA slope = 2.41*10^−5^, 95% CI in (1.09*10^−5^ 6.34*10^−5^)] than in the north [samples 5–9: Mantel correlation = 0.44, Mantel *P* = 0.086, RMA slope = 1.18*10^−5^, 95% CI in (5.38*10^−6^, 2.09*10^−5^)]. Likewise, there was no significant correlation between latitude and *H*_e_ (Pearson's *ρ *= 0.09, *P* = 0.82), and a positive but nonsignificant relationship between latitude and allelic richness (Pearson's *ρ *= 0.59, *P* = 0.09). These data suggest that the population is at or near equilibrium and that IBD theory can be used to estimate the scale of dispersal.

### Census density

In Barkley Sound, we found the number fish per 90 m^2^ transect to be 7.16 ± 1.00 (st. error) averaged over 2010 and 2011. The REEF index for Barkley Sound, BC was 2.6 (indicating abundance between 2 and 100 fish, Table 5). As explained in the methods, the REEF surveys report a weighted average of four abundance categories based on how many fish were seen throughout the dive: single (one fish), few (2–10), many (11–100), and abundant (>100). We calculated what the REEF index would be from the density transects in Barkley Sound with SCUBA, and we obtained a density score of 2.61 for our 2010 data and a SF of 66%, indicating a strong correspondence between our density estimates and REEF surveys. The REEF indexes and sighting frequencies for western Vancouver Island, the outer coast of Washington, and Oregon were similar to that reported for Barkley Sound (Table 5), indicating the fish densities were probably similar across our study area.

**Table 5:** Summary of REEF surveys for *Sebastes melanops* densities by region. The sight frequency (SF%) is the number of dive surveys in which *S. melanops* was observed (*n*) divided by the total number of dive surveys (*N*). Census density (*D*_c_) on each transect is given on a scale from 1 (rare) to 4 (abundant) – see Materials and Methods for details. The REEF index based on our SCUBA surveys in Barkley Sound was 2.61 with a SF of 66%.

Site	*N*	*n*	SF%	*D*_c_
British Columbia (BC): Western Vancouver Island (Fredericksen Pt–Cape Beale)	760	465	61.2	2.6
BC: Barkley Sound (Ucluelet– Cape Beale)	719	447	62.2	2.6
WA: Olympic Peninsula	1241	969	78.1	2.9
Oregon	903	550	60.9	2.7

The extrapolated census density from the transects gave a population size of 2.85*10^7^ (95% CI: 2.17*10^7^, 3.63*10^7^). This is close to observed estimates of the abundance of black rockfish. A mark–recapture study around Newport, OR from 2002 to 2006 (along 38.47 km of coastline) estimated the abundance of black rockfish to be 0.13*10^7^–0.21*10^7^ fish (Sampson 2007). Extrapolating the abundances from the mark–recapture study across our study range gives a slightly higher estimate of black rockfish population size of the same order of magnitude (6.00*10^7^–7.41*10^7^ fish).

### Effective density

Means and confidence intervals on *N*_e_ from the three programs are shown in Table 6. Unrealistically-large estimates of *N*_e_ (>108) were assumed to equal infinity. MIGRATE and ONeSAMP produced very different point estimates, although their confidence intervals generally overlapped. LDNe produced finite estimates in less than half the samples. LDNe and ONeSAMP failed when all individuals were pooled for analysis. For each method, we averaged the finite values of *N*_e_ over all the samples to calculate *D*_e_, assuming that the *N*_e_ of each sample was near the metapopulation *N*_e_ because of high gene flow.

**Table 6:** Mean and confidence intervals on *N*_e_ from three programs. For each program, we estimated *N*_e_ for ten datasets: one for each sampled population and one for all 572 samples pooled together. MIGRATE calculates *θ *= 4*N*_e_*μ* and we used confidence intervals on mutation rate to estimate *N*_e_ (see Materials and Methods and sResults). LDNe uses linkage disequilibrium to estimate *N*_e_ and ONeSAMP uses approximate Bayesian computation to estimate *N*_e_.

	MIGRATE			LDNE			ONeSAMP		
Population	Low CI	Mean	High CI	Low CI	Mean	High CI	Low CI	Mean	High CI
1	0	6.99	17.2	536	neg	inf	6.45E03	7.59E04	inf
2	0	7	15.6	89	342	inf	5.74E03	1.14E05	inf
3	0	7.8	16.4	127	neg	inf	2.31E03	1.42E04	5.67E05
4	0	7.8	16.4	96	545	inf	neg	2.05E06	inf
5	0	7.4	16	71	neg	inf	5.80E02	2.20E03	3.76E04
6	0	7.4	16	300	1630	inf	neg	neg	neg
7	0	8.6	17.2	95	1063	inf	1.83E03	1.31E04	1.21E06
8	0	8.2	16.8	130	neg	inf	neg	9.82E05	inf
9	0	7	15.6	148	neg	inf	8.66E02	3.54E03	4.76E04
All samples	0	7.4	16	1530	neg	inf	neg	neg	neg
Mean of finite	0	7.58	16.36	176.89	895	inf	2962	4.07E05	4.65E05
*N*_e_	0	2632	4.00E05	176.89	895	inf	2962	4.07E05	4.65E05
*D*_e_	0.00	1.94	294.12	0.13	0.66	inf	2.18	298.98	341.69

‘neg’ means the program gave a negative estimate, and ‘inf’ means the program returned infinity or an unrealistically large estimate of *N*_e_.

Point estimates for *N*_e_ from LDNe were finite in four of the nine samples and ranged from 342 to 1063 (Table 6). Confidence intervals for these estimates always included infinity, but the point estimate of *D*_e_ was extremely small (<1). We also noticed that using a different cutoff for minor allele frequency in LDNe could produce a negative or positive *N*_e_ estimate for the same locus. As LDNe seemed to fail with these data because of the numerous negative estimates and confidence intervals including infinity, we did not use them to calculate *σ*.

ONeSAMP produced finite point estimates in eight of the nine samples and ranged from 2.20∗10^3^ to 2.05∗10^6^, with 95% confidence intervals in (2962, 465 000). The point estimate of *D*_e_ from ONeSAMP equaled 299 fish/km with 95% density in (2.18, 342) (Table 6).

From the coalescent method in MIGRATE, similar estimates of θ were obtained for each sampled population and for all samples combined (Table 6). Mean *θ* across all loci equaled 7.58 with a posterior distribution with 95% density in (0, 16.36). After including confidence intervals on mutation rate, this corresponds to a point estimate of *N*_e_ equal to 2632 with 95% density in (0, 40 000). The point estimate of *D*_e_ from MIGRATE equaled 1.94 fish/km with 95% density in (0, 294).

### Dispersal distance and reserve design

**Figure 4:**
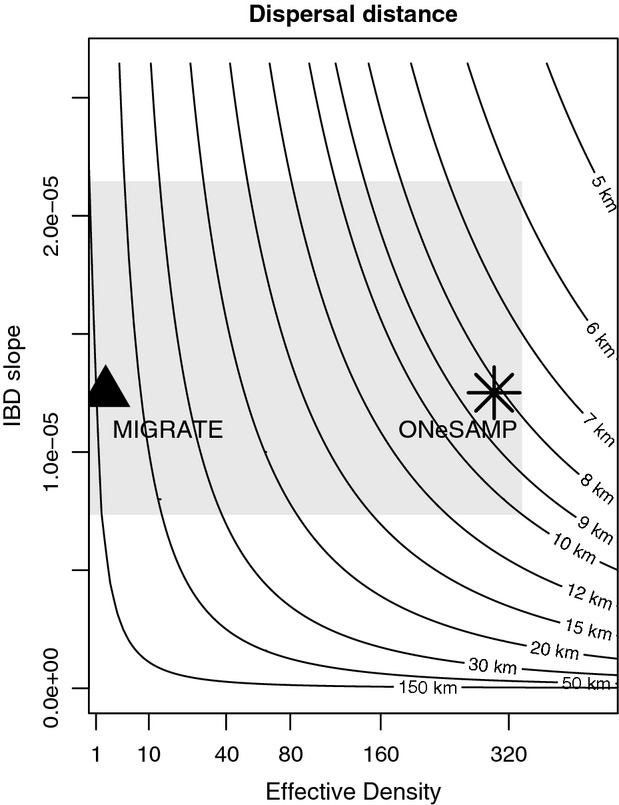
Contour lines represent the standard deviation of dispersal per generation, as a function of effective density and the Isolation by distance slope. The point estimates are shown for MIGRATE and ONeSAMP, and the gray area encompasses the 95% confidence intervals for both estimates.

We calculated point estimates for the mean dispersal distance per generation (*σ*) from MIGRATE and ONeSAMP, with confidence limits common to both programs. The point estimate on *σ* from MIGRATE was 101 km (95% CI in 6.3, 184) and from ONeSAMP was 8.2 km (95% CI in 5.8, 125) (Fig. 4).

**Figure 5:**
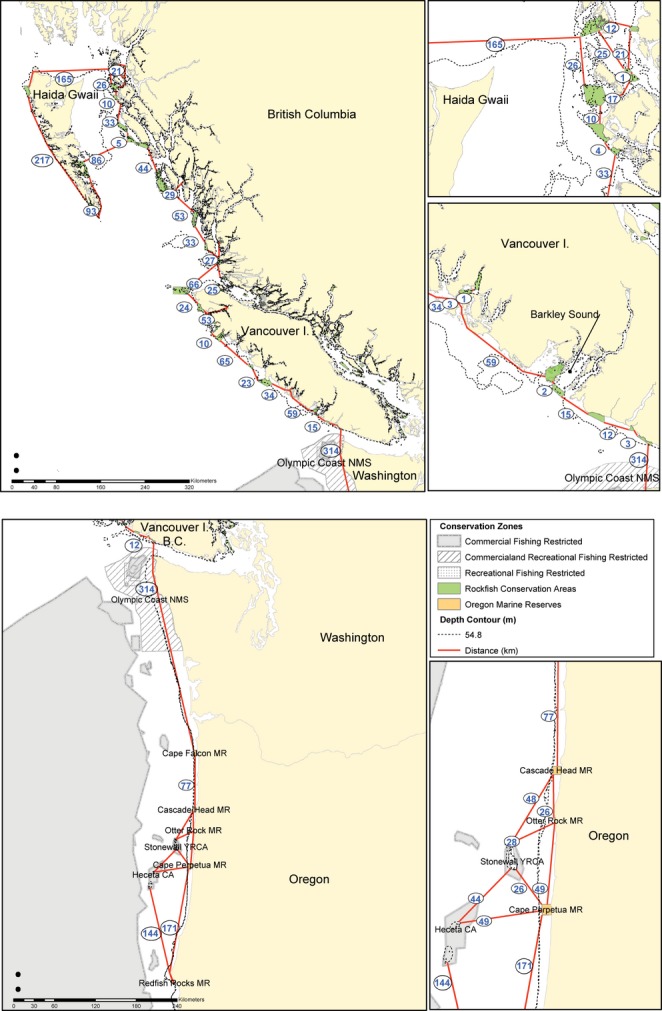
Distances between rockfish conservation areas (RCAs) that would protect black rockfish in Canada (top) and the United States (bottom); distances are in blue font inside black circles. RCAs with habitat <50 m in depth were included for analysis. Insets show detail for Haida Gwaii and Prince Rupert (upper right), Barkley Sound (center right) and central OR (bottom right). The distance between Cape Falcon MR (the northernmost nearshore reserve in the US) and Carmanah RCA (the southernmost outer-coast RCA in Canada) is 314 km.

We found that RCAs in BC are potentially connected by demographically relevant dispersal within a generation, because the distance among reserves was generally <100 km (Fig. 5, top). Likewise, RCAs in Oregon would generally be connected by dispersal within a generation because the distance among reserves was generally <100 km (Fig. 5, bottom). These two networks are unlikely to be connected by demographically relevant dispersal within a generation, however, because of a lack of RCAs along the Washington coast that would protect shallow-water rockfish species (Fig. 5, bottom). The northernmost RCA <50 m deep in the United States is the Cape Falcon Marine Reserve, and it is 314 km to the southernmost RCA in Canada at Carmanah. While dispersal is still possible through long tails on the dispersal kernel, it is unlikely to be demographically relevant, and we return to this point in the discussion.

**Figure 6:**
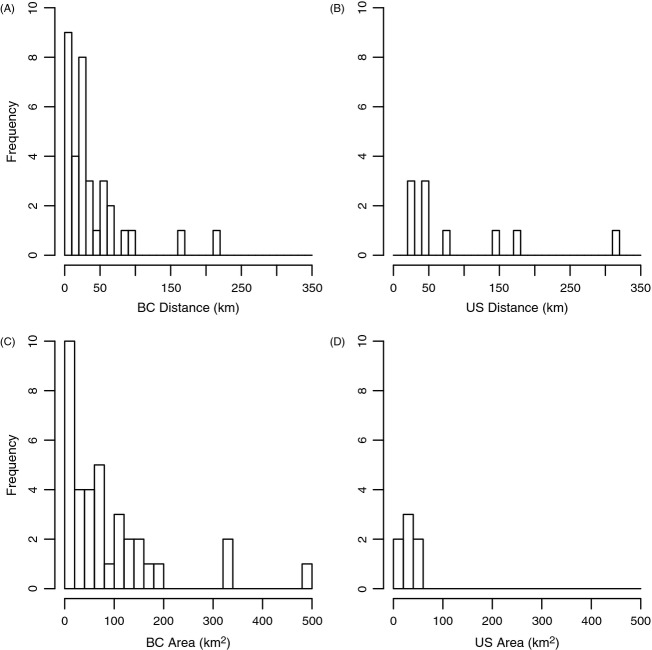
Histogram of the distance between rockfish conservation areas (RCAs) in (A) Canada and (B) the United States. Histogram of RCA areas in Canada (C) and the United States (D).

In general, there are more and larger RCAs in BC than there are in OR and WA (Fig. 6). Additionally, we were able to identify a few reserves that are likely to be isolated by dispersal from other reserves based solely on mean dispersal. In British Columbia, the reserve on the northwestern tip of Haida Gwaii (Frederick Island) is likely to be demographically isolated from other reserves on Haida Gwaii and the mainland (165 km to nearest reserve). In Oregon, Redfish Rocks Marine Reserve in the south is likely to be isolated from the network of reserves in central Oregon (144 km to nearest reserve). Redfish Rocks MR may be closer to protected areas in northern California, but this was outside of our study area.

### *N*_e_/*N*

**Figure 7:**
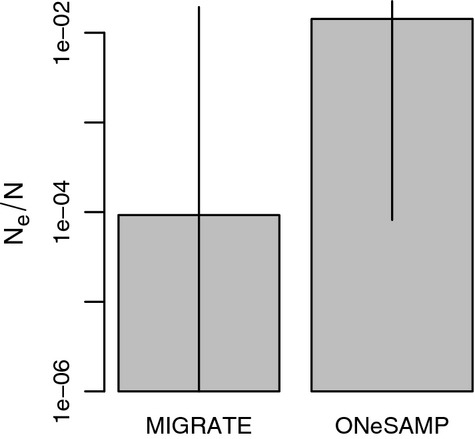
Point estimates for *N*_e_*/N* with 95% confidence intervals from MIGRATE and ONeSAMP.

The ratio of effective population size to census size was estimated to be 9.2*10^−5^ by MIGRATE and 0.014 from ONeSAMP, but confidence intervals on each estimate overlapped considerably (Fig. 7).

## Discussion

Despite low genetic structure in this system and the potential for long-distance dispersal of larvae via ocean currents, we found the scale of dispersal for black rockfish to be 6–184 km per generation. Our study adds to a growing body of evidence that low levels of genetic structure across large geographic areas are compatible with limited dispersal (Lotterhos 2012; Puebla et al. 2012). Although we obtained two different point estimates from different programs (101 km from MIGRATE versus 8.2 km from ONeSAMP), the confidence intervals for these estimates greatly overlapped. Our estimate of dispersal for black rockfish is consistent with previous studies on this species (Miller and Shanks 2004; Miller et al. 2005), although our estimate has improved accuracy because it takes into consideration the effective density in the study area. Within Canada and within Oregon, we found that the spacing between RCAs was mostly within the scale of dispersal for this species – but these reserve networks were separated by a distance of about 1.7–52 times the mean dispersal per generation. In addition, the population structure analysis further supports limited connectivity between southern Oregon (Population 1) and all other sampled populations, suggesting that realized dispersal may be even lower along the Oregon–Washington coast.

### Estimates of effective population size

A major limitation of this study that the precision *σ* depends largely on the accuracy of the *D*_e_ estimation. We found the point estimate of *N*_e_ from MIGRATE was two orders of magnitude smaller than the point estimates from ONeSAMP, although their confidence intervals overlapped substantially. Three potential (and not mutually exclusive) reasons for this are that (i) the assumed mutation rate for microsatellite markers was not representative of the loci used in this study, (ii) the historical *N*_e_ may have been much smaller because of population expansion since the last glacial maximum, or (iii) the temporal and thus spatial scale of *N*_e_ estimates is different. Our mean mutation rate was based on 61 loci from three species of fish; if the true mutation rate for the loci in this study was much smaller (i.e., 1*10^−5^), we would have obtained similar point estimates from MIGRATE and ONeSAMP. The second possibility is that *N*_e_ estimates from each program are simply reflecting different timescales: MIGRATE estimates a historical population size based on the coalescent and ONeSAMP estimates a more contemporary population size. Black rockfish are a nearshore species whose northern range was limited near the United States/Canada border by the Cordilleran ice sheet 14 000 years ago (Clague and James 2002), and the population has since expanded at least 1000 km north. The coalescent *N*_e_ may be a better estimate of the historical preglaciation population size, while the estimate from ONeSAMP reflects a more contemporary *N*_e_. If this is the case, then mean dispersal per generation may be toward the lower end of our confidence intervals (about 8 km per generation). On the third point, contemporary estimates of *N*_e_ like those from ONeSAMP may produce estimates on a more local scale (i.e., smaller) than those from a coalescent estimator like MIGRATE, but we observed the opposite pattern with our data. Still, confidence intervals on contemporary *N*_e_ were large, and in the subsequent discussion on reserve design, we assume dispersal may be as high as 184 km per generation. The accuracy of *N*_e_ estimators in populations of large census size is an area in need of more research.

We estimated *N*_e_*/N* in black rockfish to be on the order of 10^−4^ (from MIGRATE) to 10^−2^ (from ONeSAMP). The ratio of effective population size to census size is an important metric because it describes how much genetic diversity is reduced relative to the number of census individuals in the population. It is of interest to note here the trade-off between *N*_e_ and the *σ* estimated from IBD: either genetic diversity in this species is severely reduced and mean dispersal is around 100 km per generation (MIGRATE), or genetic diversity is only slightly reduced but the scale of dispersal is <10 km per generation (ONeSAMP). Either case could be used as an argument for greater protection of the species.

A ratio of *N*_e_/*N *≪ 1 may be due to several nonideal conditions such as multiple paternity and overlapping generations (Lotterhos 2011), fluctuating population size (Vucetich et al. 1997), spatial variance in habitat quality (Nunney 1999), and high variance in reproductive success among individuals (Berkeley et al. 2004a; Hedrick 2005). In a recruitment study of black rockfish in Barkley Sound, BC, large fluctuations in recruitment were observed from year-to-year, along with genetic signatures of high variance in reproductive success such as sibships and low effective number of breeders (Lotterhos and Markel 2012) – suggesting that oceanographic uncertainty may be partially responsible.

### Genetically relevant versus ecologically relevant connectivity

There is an important distinction in how migration of individuals affects the genetic structure versus the demographics of a population, and this distinction has important implications for how our results are interpreted in light of reserve network design. Genetic connectivity depends primarily on the absolute number of dispersers among populations, whereas demographic connectivity depends on the relative contributions of immigrants and local recruitment to population growth rates (Lowe and Allendorf 2010).

From the perspective of gene flow, just a few migrants per generation can greatly reduce estimates of genetic differentiation (Slatkin 1987; Waples 1998; Palumbi 2003). In an island model, as few as one migrant per generation is enough genetic exchange to prevent large genetic differences from accumulating (Slatkin 1987). In addition, small levels of gene flow may be enough to reduce the harmful effects of inbreeding or genetic drift, and to increase integrity of the species through the spread of selectively advantageous alleles (Lowe and Allendorf 2010). Therefore, the reserve spacing in the United States and Canada is likely to be sufficient to for the exchange of alleles between reserves, based on the genetic diversity introduced by a few long-distance dispersers.

A few migrants per generation, however, is a small amount of demographic exchange, and will not impact population dynamics on ecological timescales. Here, we refer to ‘long-distance dispersal’ to be defined relative to the mean dispersal distance (Kinlan and Gaines 2003). Although the critical number of recruits required to be demographically important has not been determined, a few long-distance dispersers will not crucially impact the demography of the population (Kinlan and Gaines 2003). To sustain a heavily fished population, recruitment to the local population must be subsidized from a reserve or upstream source on the order of the prefishery recruitment rates, which will not be sustained by a few immigrants (e.g., Cowen et al. 2003). Likewise, reserve design is more concerned with the buildup of multiple age classes over time, and not the immigration of a few individuals on sporadic timescales (Gaines et al. 2010).

Although the overall *F*_ST_ in black rockfish was low (and genetic exchange in this population is therefore high via a few long-distance migrants), IBD theory reflects average dispersal distance per generation and is therefore closer to ecologically-relevant dispersal distances. The IBD slope is expected to be heavily affected by dispersal during the several most recent generations, and is not affected by rare dispersal events (Rousset 1997). The distance between Cape Falcon MR (the northernmost nearshore reserve in the US) and Carmanah RCA (the southernmost outer-coast RCA in Canada) is 314 km – and for black rockfish it could take 1.7–52 generations for an ecologically-meaningful number of migrants to be exchanged between these two areas. As rockfish species are slow to reproduce (5–10 years to reach maturity), it may take decades for demographically relevant migrant exchange to occur.

### Isolation by distance, subtle genetic structure, and oceanographic features

The IBD slope that we measured in black rockfish is within the range of that observed in other rockfish species. Table 7 summarizes the published IBD slopes in *Sebastes* spp., sorted from smallest to largest IBD slopes. Most of these studies also inferred *σ* from the IBD slope but did not estimate *D*_e_ and instead examined a range of values. As *σ* approaches infinity as *D*_e_ approaches zero (eqn 1), it is important to have a lower limit on *D*_e_ to infer an upper limit on σ. Our lowest confidence interval was *D*_e_ = 1, but other studies did not consider such a low *D*_e_ (e.g., *D*_e_ = 10 was the lowest considered by other studies, Table 7). Therefore, published estimates of σ in rockfish may be biased lower than they are in reality (Table 7). Although all other studies employed OLS regression (which may underestimate the true regression slope – see ‘Materials and Methods: Estimation of IBD slope’), the resulting estimate of *σ* will be less sensitive to small changes in the IBD slope than an order of magnitude change in *D*_e_ (eqn 1). As our upper confidence interval of *σ* is based on *D*_e_ = 1, this is a conservative estimate of the upper limit on mean dispersal of black rockfish larvae. We also note that sampling design can affect the inference of *σ*. Two studies on copper rockfish found three orders of magnitude difference in the IBD slope (Buonaccorsi et al. 2002; Johansson et al. 2008), but these studies differed in the regions sampled and number of sites sampled. In our study, we limited our sampling design (and we limit our subsequent discussion) to black rockfish populations on the outer coast.

**Table 7:** Summary of other studies in rockfish (*Sebastes* spp.) that use microsatellite DNA to measure the IBD slope in rockfish. Studies are sorted from the lowest to the highest isolation by distance (IBD) slope. Columns indicate the observed correlation from a Mantel test or *R*^2^ from regression (Mantel *r* or *R*^2^), the *P*-value of the Mantel test (*P*-value), the distance between the furthest samples (Scale), the estimate of the IBD slope, the range of *σ* reported, and the span of effective densities (*D*_e_ span) that were used to calculate σ (n.r. = not reported). Most studies did not estimate *D*_e_ from genetic data and instead examined a range of values. As *D*_e_ is inversely related to *σ*, only examining a range of high values of *D*_e_ may give a much smaller estimate of dispersal than is true in reality. Two studies on copper rockfish found three orders of magnitude difference in the IBD slope – Buonaccorsi et al. (2002) sampled from California to Haida Gwaii and Johansson et al. (2008) sampled from southern California to Washington.

Study	Species	Common name	Mantel *r* or *R*^2^	Mantel *P*-value	Scale (km)	IBD slope	*σ* (km)	*D*_e_ span
Siegle et al. (2013)	*Sebastes ruberrimus*	Yelloweye rockfish	0.099	0.27	1500	0.0000005	n.a.	n.a.
Buonaccorsi et al. (2004)	*Sebastes rastrelliger*	Grass rockfish	0.079	0.019	1300	0.000002	1–35	10–10 000
Gharrett et al. (2012)	*Sebastes polyspinis*	Northern rockfish	n.r.	0.007	1900	0.000002	2–30	133–13 277
Gomez-Uchida and Banks (2005)*	*Sebastes crameri*	Darkblotched rockfish	0.2	0.041	1000	0.000004	1.72	20 706
Johansson et al. (2008)	*Sebastes caurinus*	Copper rockfish	0.42	0.0009	2200	0.000006	n.r.	n.r.
Palof et al. (2011)	*Sebastes alutus*	Pacific Ocean Perch	0.45	0.004	3400	0.000007	7–70	129–12 875
This study	*Sebastes melanops*	Black rockfish	0.57	0.005	1400	0.00001	18–184	1–50
Buonaccorsi et al. (2005)	*Sebastes auriculatus*	Brown rockfish	0.28	0.045	1000	0.00003	1–20	10–10 000
Hyde and Vetter (2009)	*Sebastes miniatus*	Vermillion rockfish	0.44	0.001	2100	0.0001	0.7–22.5	10–10 000
Buonaccorsi et al. (2002)†	*Sebastes caurinus*	Copper rockfish	0.82	0.041	2100	0.008	1–40	10–10 000
Hess et al. (2011)‡	*Sebastes flavidus*	Yellowtail rockfish	0.78	0.0001	2500	0.017	n.r.	n.r.

* Estimate excluding small sample sizes but not pooling samples.

† Scale of dispersal reported in Buonaccorsi et al. (2005).

‡ Estimate from microsatellites. Samples spanned a regional faunal break and this resulted in a high IBD slope.

We found three lines of evidence that supported the hypothesis of limited connectivity between southern Oregon (Port Orford, population 1) and the rest of the sampled range: (i) the pairwise *F*_ST_ ‘s showed that population 1 was significantly differentiated from all other sampled populations; (ii) population 1 was an outlier in the PC analysis, and (iii) the IBD slope was higher when only sites in OR and WA were included. These lines of evidence are in agreement with a previous studies on black rockfish (Miller et al. 2005), grass rockfish Buonaccorsi et al. (2004), copper rockfish (Johansson et al. 2008), and vermillion rockfish (Hyde and Vetter 2009).

So what are the putative barriers to dispersal within the Oregonian province? In other rockfish species, the distribution of genetic diversity has been partitioned by large-scale oceanographic barriers (Rocha-Olivares and Vetter 1999; Hyde and Vetter 2009), and regional faunal breaks (Hess et al. 2011; Palof et al. 2011). For black rockfish on the Oregon coast, we discuss three (not mutually exclusive) possibilities: (i) unsuitable sand habitat, (ii) a transition in upwelling dynamics that affects reproductive success, and (iii) the presence of a geographical headland that restricts gene flow. Johansson et al. (2008) suggest that long stretches of sand along the Oregon coastline could act as barriers to dispersal for copper rockfish. Copper rockfish, however, have a much shorter pelagic period than black rockfish (Table 8) and are expected to release larvae nearshore and to have more limited dispersal (Johansson et al. 2008; Markel 2011). Therefore, black rockfish larvae may be less susceptible than copper rockfish larvae to sand barriers that are large enough to decrease the likelihood of survival.

**Table 8:** Summary of rockfish species that occur from nearshore to the shallow-shelf, and whose range includes part of our study area. The depth range in which they have been found is indicated, as well as information on mean depth if available. We also report the range on the pelagic larval duration (PLD) and the mean, if known.

Species	Common name	Depth range, m (mean)	PLD (mean)	Citations
*Sebastes melanops*	Black	0–55 (~20 m)	42–105 days (75 days)	1, 2, 3, 4, 5
*Sebastes auriculatus*	Brown	0–120	2.5–3 months	1
*Sebastes caurinus*	Copper	0–90	32–88 days (56 days)	1, 3
*Sebastes emphaeus*	Puget Sound	3–366	Unknown?	1
*Sebastes maliger*	Quillback	0–274	Unknown?	1
*Sebastes mystinus*	Blue	0–90	3–5 months	1
*Sebastes nebulosus*	China	3–128	Unknown?	1

References: (1) Love et al. (2002); (2) Lotterhos and Markel (2012); (3) Markel (2011); (4) Johnson et al. (2003); (5) Parker et al. (2007).

Another likely possibility is a transition in the upwelling regions of the California Current. According to Parrish et al. (1981), the California Current system can be divided into four compartments based on mean geostropic flow, Ekman transport, and wind stress curl. From south to north, these compartments are: south Baja California; the southern California Bight; Point Conception, CA to Cape Blanco, OR; and Cape Blanco to Vancouver Island, BC. The region from Point Conception to Cape Blanco is known as the region of maximum upwelling, and the region from Cape Blanco to Vancouver Island has weaker upwelling that is of shorter duration (Parrish et al. 1981). Cape Blanco, Oregon is located about 20 km north of Port Orford (population 1). Interestingly, *Sebastes* spp. have a local minimum in abundance within the maximum upwelling region (Parrish et al. 1981). The black rockfish pelagic period is between mid-February to early-May. During this time north of Cape Blanco, surface Ekman transport is predominately onshore, but sites south of Cape Blanco will experience higher upwelling in the early spring. Strong upwelling is thought to increase the offshore transport and mortality of fish larvae and has been shown to cause sweepstakes-like recruitment of young-of-the-year black rockfish (as indicated by high number of sibships and low effective number of breeders, Lotterhos and Markel 2012). If upwelling plays an important role in both dispersal and the distribution of reproductive success in black rockfish populations located in southern Oregon, it may explain the lower connectivity between this region and sites to the north. As a third and related reason for reduced dispersal, Cape Blanco is a geographical headland along the coast, and such promontories are known to cause retention zones that entrain rockfish larvae and may form discrete barriers to gene flow (Morgan and Botsford 1998; Wing et al. 1998).

### Reserve design for black rockfish and other nearshore rockfishes in United States and Canada

The design of a reserve network should consider the conservation and fishery management goals. The criteria for reserve siting may include any of the following: representation of biogeographic regions, maximization of species diversity, maximization of habitat heterogeneity (including adult versus nursery habitats), and the representation of retention zones (Botsford et al. 2003). These criteria, however, do not specify how large reserves should be, or how far apart they should be spaced. It is generally agreed that a reserve network should be self-sustaining (Carr et al. 2003), but how this is achieved also depends on fishery management goals. For example, if the goal is to protect the spawning stock biomass of a particular spatial area, than individual reserves should be large relative to mean dispersal of the species so that the reserve is self-sustaining. Alternatively, if the goal is to subsidize an external fishery then smaller reserves should be designed to be connected by dispersal (Botsford et al. 2003; Carr et al. 2003; Sale et al. 2005). Generally, larval dispersal has a profound influence on the predicted size and spacing of reserves (Botsford et al. 2003), but predictions get more complicated when other aspects of uncertainty are included such as fishing effort, life-history traits, and home-range size (Walters et al. 2007; Moffitt and White 2011).

In the subsequent discussion, we assume that a reserve network for rockfishes in the United States and Canada should be designed to maximize population growth of all members of the genus and to maximize subsidy to the fishery. A comprehensive RCA network should therefore meet the following minimum criteria (based on the studies cited above and specific recommendations for rockfishes by Starr 1998):

1. Reserves should be located in all upwelling bioregions;
2. Reserves should contain representative habitats for different species and life stages;
3. A network should be self-sustaining regardless of the populations outside of it, and therefore, reserves should be spaced by at least the mean dispersal distance of the lowest dispersing species; and
4. Reserves should be distributed along the coast at various distances from headlands to account for retention and/or high variance in reproductive success caused by uncertainty in oceanographic conditions (for a discussion of this point see also Larson and Julian 1999).

Our population structure analysis suggested that there is a genetic break near Cape Blanco, Oregon, and we discussed how this location corresponds to a shift in upwelling dynamics (criteria *i*) that may affect reproductive success and larval retention (criteria *iv*). There is only one reserve located south of Cape Blanco at Redfish Rocks, and this reserve is located 144–171 km from the nearest reserves at Hecata and Cape Perpetua – distances near the upper limit on our estimate of mean dispersal for black rockfish. Given the lower genetic connectivity observed in many *Sebastes* spp. in this area, it is likely that criterias *i* and *iv* are violated in the southern part of Oregon.

In Washington, there are no shallow-water reserves that would protect nearshore rockfish species, despite the presence of the Olympic Coast National Marine Sanctuary (OCNMS). Although there are some deep water RCAs in Washington, these areas are designed to protect yelloweye (*Sebastes ruberrimus*) rockfish, and are outside the depth range of nearshore-to-shallow-shelf species like black rockfish. Many other rockfish species share this shallow depth preference (Table 8, Love et al. 2002; Berntson and Moran 2009) and are unlikely to be protected by the deepwater reserves. The lack of nearshore reserves in Washington therefore violates criteria *ii* for nearshore rockfish species. Likewise, the lack of reserves in this area also results in a violation of criteria *iii* for black rockfish, when considering the network of reserves in United States and Canada as a whole.

Black rockfish are just one of a suite of species that are protected by RCAs, and optimal reserve design for a suite of species becomes more complicated. In a review dispersal distances for a variety of taxa, Shanks et al. (2003) found a bimodal distribution of dispersal, with a range for long-distance dispersers on the order of 20–200 km/year. They suggested that the minimum spacing between reserves should be approximately the mean dispersal for the lowest dispersing species, assuming that larvae dispersing longer distances can settle into several reserves along the coast. As black rockfish have a longer pelagic larval duration (PLD) than some of the other nearshore rockfishes (Table 8), our analysis of reserve connectivity is probably conservative (because connectivity would probably be less for other *Sebastes* species).

### Dispersal models and reality

Our analysis was limited because it focused mainly on dispersal distance, and IBD theory assumed a well-mixed larval pool with uniform dispersal and ignored other important aspects of uncertainty. The assumption of a single dispersal distribution that applies across an entire marine landscape is unlikely to be justified given the asymmetry of oceanographic currents and the potential effect of larval behavior on dispersal (Cowen et al. 2000, 2006; Mitarai et al. 2008; Siegel et al. 2008; Treml et al. 2008; Alberto et al. 2011). In the presence of asymmetric dispersal along a coastline caused by an advective current, there is an increase in the total area of reserve required for persistence because advection reduces self-retention (Botsford et al. 2001, 2009). Under this type of source-sink dynamics, fisheries yield is predicted to be greater when the source population is placed inside a reserve (Fogarty and Botsford 2007; Gaines et al. 2010). Yet, it is difficult to have an *a priori* prediction for asymmetric dispersal in *S. melanops* and other rockfish species, as their PLD spans a seasonal switching from predominately downwelling and southerly winds that drive northward transport, to predominately upwelling and northerly winds that drive southward transport (OKs and Markel 2012). Drogues (trackers passively dispersed by ocean currents) released in January–February along the Washington Olympic peninsula show net northerly transport (Park et al. 2007). Black rockfish larvae experience northerly transport early in their PLD, and then southerly transport later in their PLD, and this could result in minimal net alongshore transport (e.g., the ‘marine drift paradox,’ Shanks and Eckert 2005). In addition, rockfish larvae are capable swimmers shortly after parturition (Fisher et al. 2007), and early onset of active larval movement can play a large role in mediating dispersal potential (Cowen et al. 2006). Estimating asymmetry in dispersal from genetic data is complicated because gene flow will be affected by both density and asymmetric movement of propagules (Kirkpatrick and Barton 1997; Eckert et al. 2008). Understanding the interaction between density and asymmetric movement of propagules on the estimation of asymmetric gene flow in advective marine systems – and the different ecological and evolutionary implications of this interaction – is an area in need of additional research.

## Conclusions

We found that the average dispersal per generation in black rockfish was smaller than the distance between reserve networks that protect this species in Canada and Oregon. From the perspective of gene flow, the distance between reserves is probably sufficient to maintain genetic integrity of the species. But from the perspective of demographic connectivity, some areas of the network are unlikely to experience demographically relevant exchange. If additional reserves were to be implemented, we suggest that they be located in southern Oregon and on the Washington coast. Additional reserves in this area could improve connectivity among RCAs in both countries. Marine reserves play an important role in not only enhancing biodiversity (Russ and Alcala 2011) and maintaining species biomass and abundance (Cote et al. 2001; Lester et al. 2009), but also in maintaining and protecting older age classes of fish (Berkeley et al. 2004b). These older fish may play an important ecological role by contributing disproportionally to population productivity and population growth (Berkeley et al. 2004b; Berkeley 2006) and may play an important evolutionary role by reducing the genetic consequences of fisheries-induced evolution (Miethe et al. 2010), or by harboring genetic diversity that may be adaptive under climate change.

Although models have heuristic value in understanding the processes that determine the success of a reserve network, careful monitoring and evaluation will be required to evaluate RCAs as a tool for conservation and increasing fishery yields (Hilborn et al. 2004; Gaines et al. 2010). As discussed above, there are a wide variety of life-history strategies for rockfishes with PLDs ranging from several weeks to several months as well as differences in timing of larval release (Love et al. 2002), which may complicate the evaluations of RCAs. Ultimately, the importance of each reserve's contribution to network persistence will depend on rockfish density, age structure, and fine-scale patterns of dispersal caused by oceanographic currents and features such as retention zones (e.g. White et al. 2010). To further evaluate the performance of RCAs along the west coast of United States and Canada, spatially explicit data on egg or larval production (which depends on adult age structure, fecundity, and density of rockfishes inside reserves) will need to be integrated with more detailed models of oceanographic and genetic connectivity for different species inside reserves (Botsford et al. 2009).

## Data archiving

All data, scripts, and results from this study have been deposited in the Dryad Digital Repository: 10.5061/dryad.m0p56 (Lotterhos^2013^).
