# Supplementary material for: Evaluation of rockfish conservation area networks in the United States and Canada relative to the dispersal distance for black rockfish (Sebastes melanops)
Source: Evol Appl. 2013 Nov 4;7(2):238–59. doi: 10.1111/eva.12115 (PMC3927886; doi:10.1111/eva.12115)

1 Supplementary Materials

1.1 STRUCTURE methods

We tested three parameter sets ("Set 1", "Set 2", and "Set 3") which only differed in the LOCPRIOR model. The LOCPRIOR model uses a user-defined sampling location to modify the prior distribution for each individual's population assignment, and typically allows genetic structure to be detected at lower levels of divergence (Hubisz et al. 2009). In Set 1, we used the sampling location shown in Figure 1 as the LOCPRIOR for each individual. In Set 2, we set the samples from Oregon (populations 1–2 in Figure 1) as LOCPRIOR=1 and all other samples as LOCPRIOR=2. This second set was chosen because previous studies on rockfish found a genetic break in Oregon. In Set 3, we set population 1 as LOCPRIOR=1, population 2 as LOCPRIOR=2, populations 3-8 as LOCPRIOR=3, and population 9 as LOCPRIOR=4. This set allowed for the possibility of an oceanographic break between Vancouver Island and Haida Gwaii, where the Subarctic Current splits into the northward-flowing Alaska current and the southward-flowing California current (although see discussion about timing of larval release relative to oceanographic events in the section "Study system and species"). These three LOCPRIOR models are summarized in Supplementary Table S1. For all sets, the length of the burn-in period was set to 100,000 with 100,000 MCMC reps after burn-in. We used the admixture model with correlated allele frequencies among populations and a prior of F_{ST} equal to 0.002 (see Results). All other parameters were default.

We ran five replicates of each parameter set for K=1 to K=9, and used the website STRUCTURE HARVESTER (Earl and vonHoldt 2012) to summarize the results. In inferring K, we used a combination of three methods: (i) we graphed the natural logarithm of the likelihood of the data given K (L(K)) as suggested by Pritchard et al. (2000), (ii) we graphed ΔK as a function of K as suggested by Evanno et al. (2005), and (iii) we examined

the bar plot(s) showing individual assignments for K's that had high likelihoods by methods (i) and (ii). Since population structure was weak in our data, special attention was given to the comparison of K = 1 versus greater values, as the Evanno method is not capable of performing this comparison. We used CLUMMP (Jakobsson and Rosenberg 2007) to summarize replicate runs for each K, and used an R script to plot individual assignments for the given K.

Table S1: Summary of LOCPRIOR models used in the STRUCTURE analysis.

Population	Set 1	Set 2	Set 3
1	1	1	1
2	2	1	2
3	3	2	3
4	4	2	3
5	5	2	3
6	6	2	3
7	7	2	3
8	8	2	3
9	9	2	4

1.2 STRUCTURE results

In PRIORLOC Set 1, K=1 clearly had the highest L(K), although bumps occurred in the ΔK plots at K=7 and K=9 (Supplementary Figure S1). In PRIORLOC Sets 2 and 3, likelihoods were similar for K=1 to K=4, and the ΔK plot showed a bump at K=2 and K=4 (Supplementary Figures S2-S3). An examination of the barplots for K=2 to K=4 from Set 3 showed no strong genetic structure, but a subtle pattern of isolation by distance (Supplementary Figure S4).

References

Earl, D. A., and B. M. vonHoldt. 2012. STRUCTURE HARVESTER: a website and program for visualizing STRUCTURE output and implementing the Evanno method. Conservation Genetics Resources 4:359–361.

Evanno, G., S. Regnaut, and J. Goudet. 2005. Detecting the number of clusters of individuals using the software STRUCTURE: a simulation study. Molecular Ecology 14:2611–2620.

Hubisz, M., D. Falush, M. Stephens, and J. Pritchard. 2009. Inferring weak population structure with the assistance of sample group information. Mol Ecol Resour 9:1322–1332.

Jakobsson, M., and N. A. Rosenberg. 2007. CLUMPP: a cluster matching and permutation program for dealing with label switching and multimodality in analysis of population structure. Bioinformatics 23:1801–1806.

Pritchard, J. K., M. Stephens, and P. Donnelly. 2000. Inference of population structure using multilocus genotype data. Genetics 155:945–959.

Figure S1: L(K) and ΔK plots from the STRUCTURE analysis with the Set 1 LOCPRIOR model.

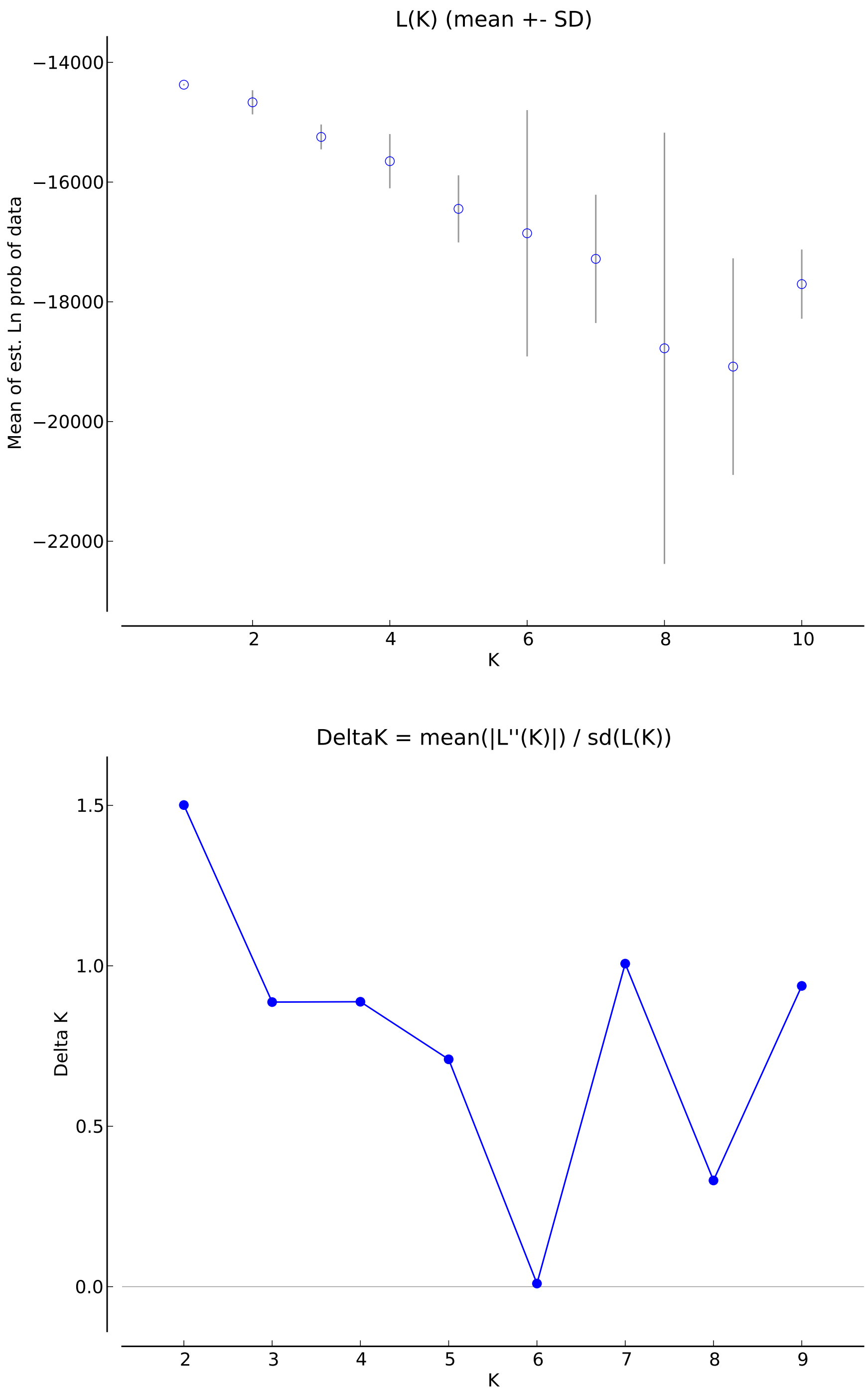

Figure S2: L(K) and ΔK plots from the STRUCTURE analysis with the Set 2 LOCPRIOR model.

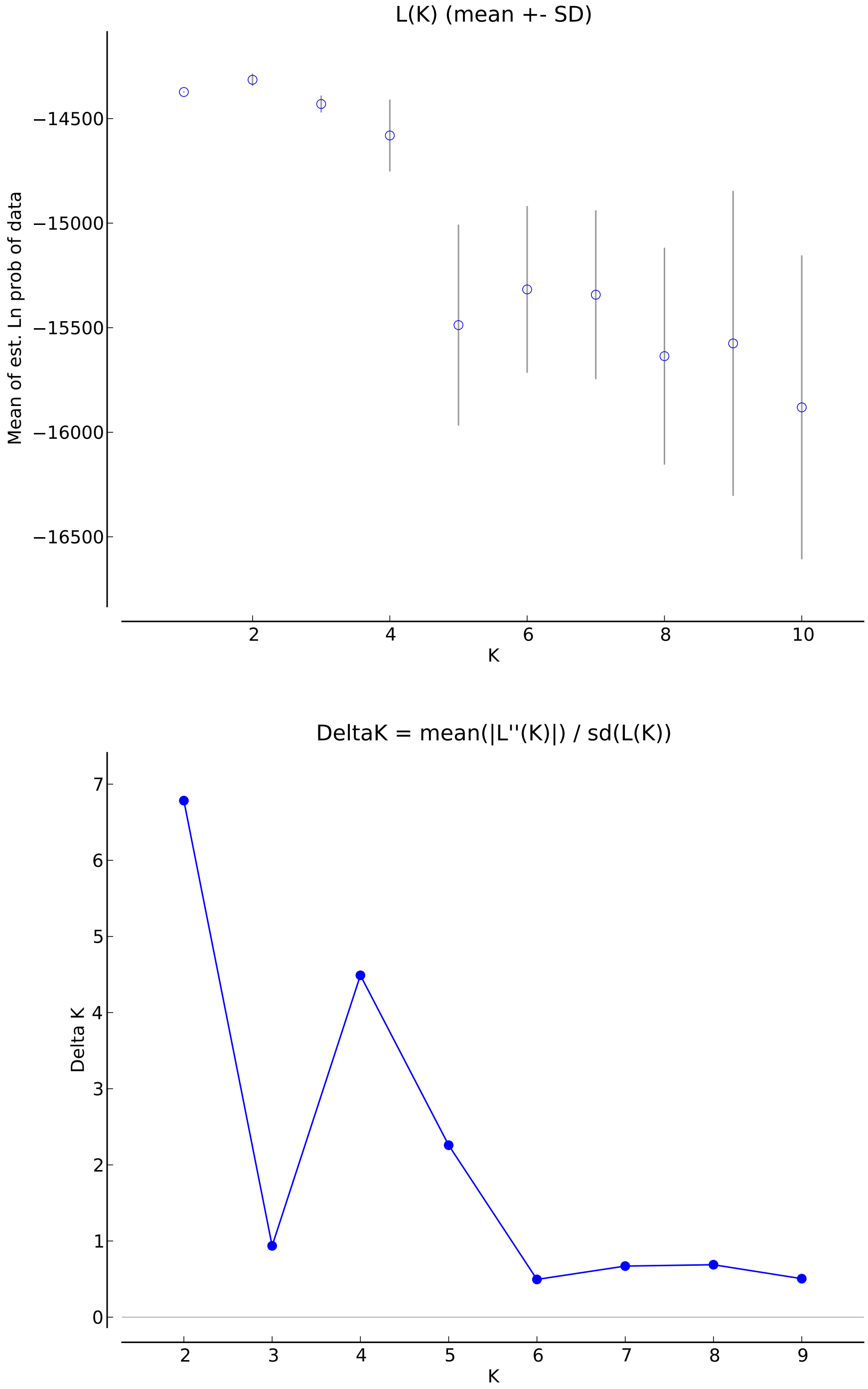

Figure S3: L(K) and ΔK plots from the STRUCTURE analysis with the Set 3 LOCPRIOR model.

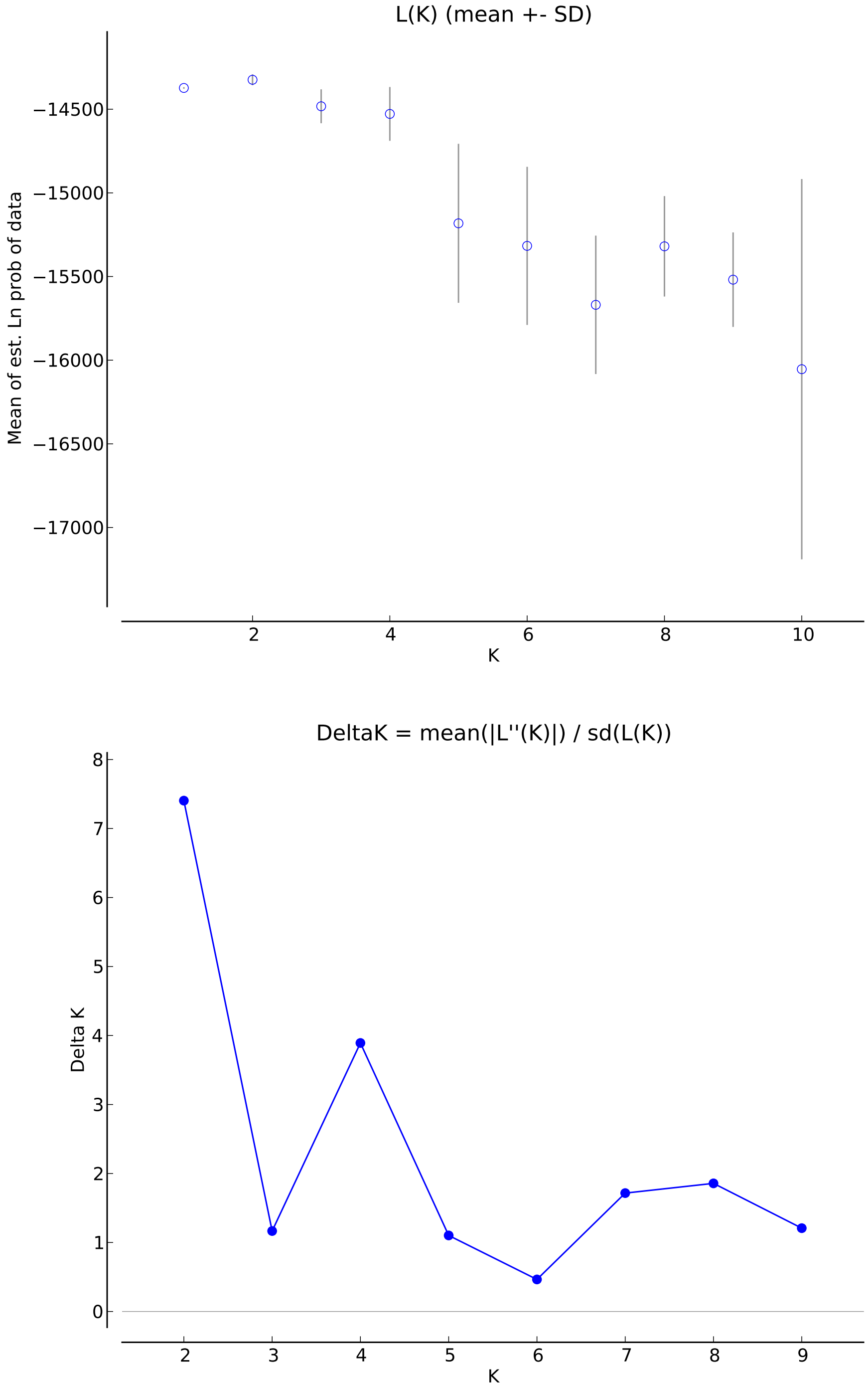

Figure S4: Bar plots for K=2, K=3, and K=4 from the Set 3 STRUCTURE analyses. The six individuals with "mixed" ancestry tended to have more than one rare microsatellite allele.

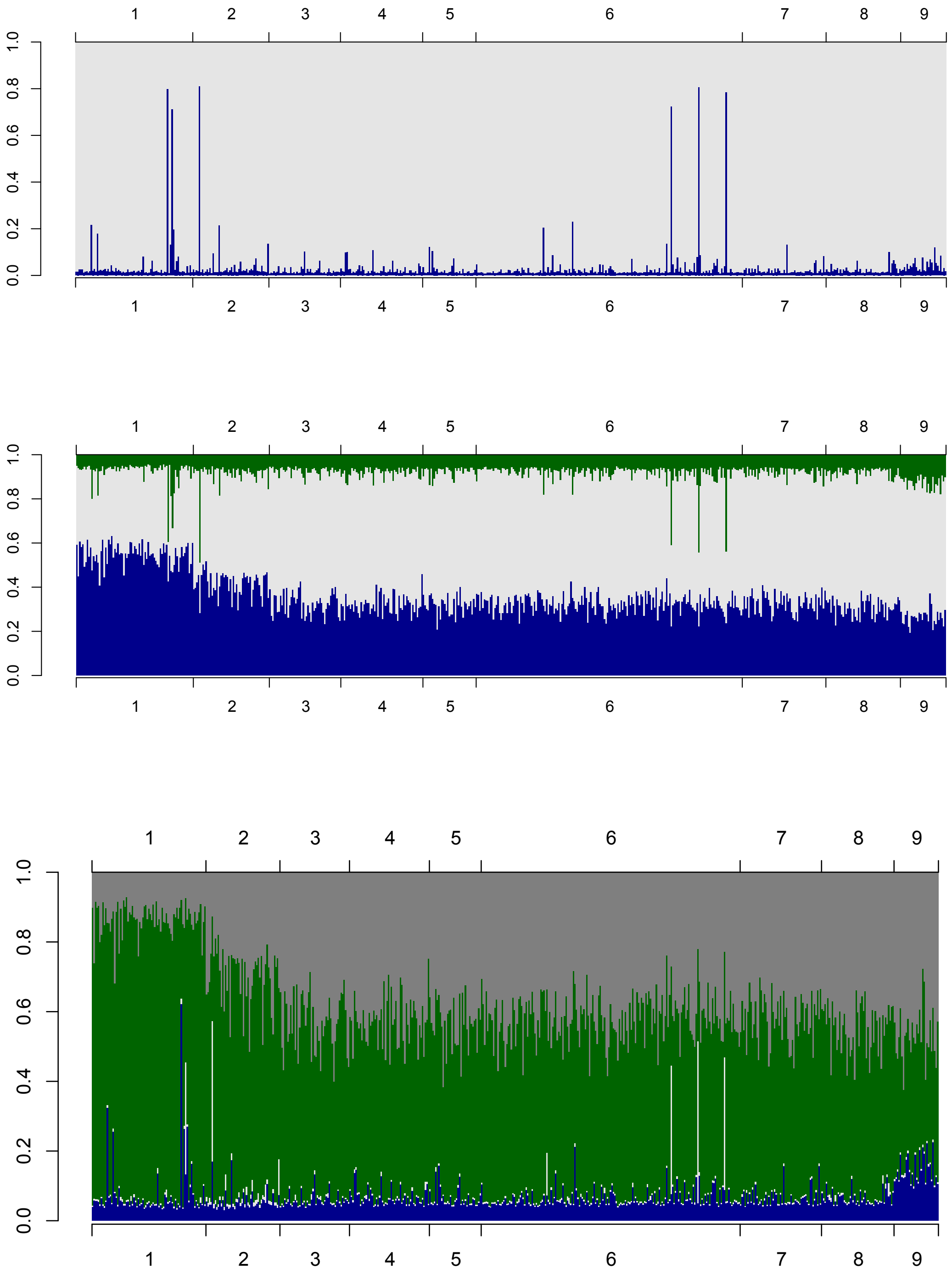

Supplement: Supplementary file 1 — Data S1. Materials and methods. [file eva0007-0238-sd1.pdf]
